# Molecular pathological classification of colorectal cancer

**DOI:** 10.1007/s00428-016-1956-3

**Published:** 2016-06-20

**Authors:** Mike F. Müller, Ashraf E. K. Ibrahim, Mark J. Arends

**Affiliations:** 1 Division of Pathology, Centre for Comparative Pathology, Edinburgh Cancer Research Centre, Institute of Genetics & Molecular Medicine, Western General Hospital, University of Edinburgh, Crewe Road South, Edinburgh, EH4 2XR UK; 2 Department of Pathology, Addenbrooke’s Hospital, University of Cambridge, Hills Road, Cambridge, CB2 0QQ UK; 3 Bedford Hospital NHS Trust, Viapath Cellular Pathology, Kempston Road, Bedford, MK42 9DJ UK

**Keywords:** Colorectal, Cancer, Polymerase epsilon, Ultramutant, Hypermutant, Defective mismatch repair, Microsatellite instability, Chromosomal instability, Mutation, Somatic copy number alterations, Consensus molecular subtypes, The Cancer Genome Atlas, Serrated pathway

## Abstract

Colorectal cancer (CRC) shows variable underlying molecular changes with two major mechanisms of genetic instability: chromosomal instability and microsatellite instability. This review aims to delineate the different pathways of colorectal carcinogenesis and provide an overview of the most recent advances in molecular pathological classification systems for colorectal cancer. Two molecular pathological classification systems for CRC have recently been proposed. Integrated molecular analysis by The Cancer Genome Atlas project is based on a wide-ranging genomic and transcriptomic characterisation study of CRC using array-based and sequencing technologies. This approach classified CRC into two major groups consistent with previous classification systems: (1) ∼16 % hypermutated cancers with either microsatellite instability (MSI) due to defective mismatch repair (∼13 %) or ultramutated cancers with DNA polymerase epsilon proofreading mutations (∼3 %); and (2) ∼84 % non-hypermutated, microsatellite stable (MSS) cancers with a high frequency of DNA somatic copy number alterations, which showed common mutations in *APC, TP53*, *KRAS*, *SMAD4*, and *PIK3CA*. The recent Consensus Molecular Subtypes (CMS) Consortium analysing CRC expression profiling data from multiple studies described four CMS groups: almost all hypermutated MSI cancers fell into the first category CMS1 (MSI-immune, 14 %) with the remaining MSS cancers subcategorised into three groups of CMS2 (canonical, 37 %), CMS3 (metabolic, 13 %) and CMS4 (mesenchymal, 23 %), with a residual unclassified group (mixed features, 13 %). Although further research is required to validate these two systems, they may be useful for clinical trial designs and future post-surgical adjuvant treatment decisions, particularly for tumours with aggressive features or predicted responsiveness to immune checkpoint blockade.

## Introduction

Colorectal cancer (CRC) is the third most common cancer in men and the second most common cancer in women, accounting for about 700,000 deaths per year [1]. The majority of 70–80 % of CRC are sporadic, while around 20–30 % of CRC have a hereditary component, due to either uncommon or rare, high-risk, susceptibility syndromes, such as Lynch Syndrome (LS) (3–4 %) and familial adenomatous polyposis (FAP) (∼1 %) [2], or more common but low-risk alleles. Some of the latter, such as *Shroom2*, have been identified by genome-wide association studies (GWAS) [3]. A small subset of about 1–2 % of CRC cases arises as a consequence of inflammatory bowel diseases [4].

**Fig. 1:**
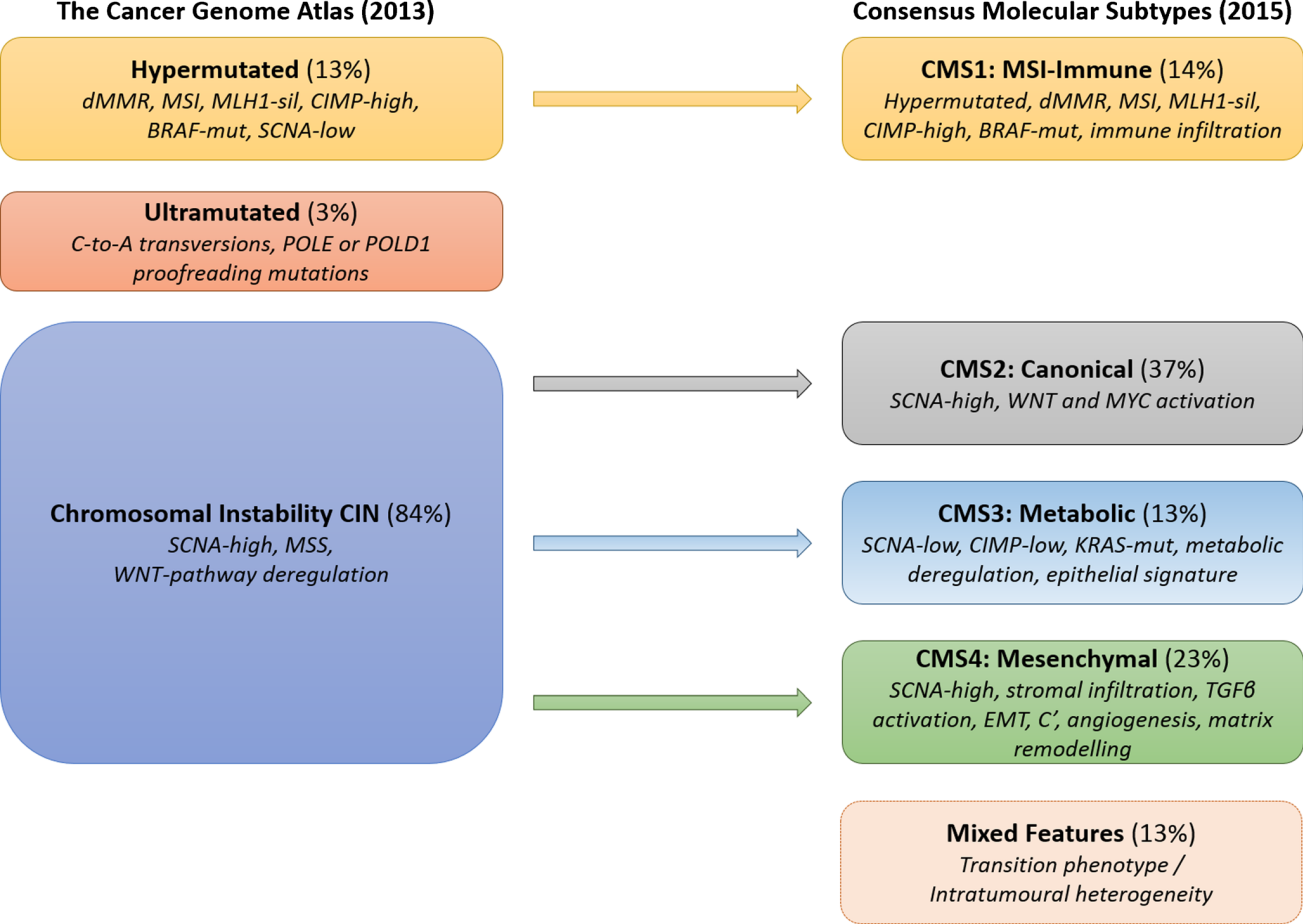
Molecular classification systems for colorectal cancers. *On the left* is a representation of The Cancer Genome Atlas integrated molecular classification of colorectal cancers into three groups: (*1*) ∼13 % hypermutated tumours with microsatellite instability due to defective mismatch repair, usually caused by *MLH1* silencing via promoter hypermethylation, with the dMMR pathway causing a hypermutated phenotype resulting from failure to recognise and repair DNA mismatches or insertions/deletions; 80–90 % of sporadic hypermutated cancers have *BRAF* V600E (or similar) mutations; (*2*) ∼3 % ultramutated tumours with DNA Polymerase Epsilon or Delta 1 (*POLE* or *POLD1*) exonuclease domain (proofreading) mutations (EDM), with the malfunctioning enzyme introducing incorrect nucleotides during DNA replication, resulting in an ultramutated phenotype; (*3*) ∼84 % CIN tumours with a high frequency of DNA SCNAs, a low mutation rate (<8/Mb), microsatellite stability (MSS) and deregulation of the WNT pathway most frequently by *APC* mutation. *On the right* is a representation of the consensus molecular subtypes (CMS) expression signature-based classification with four CMS groups—CMS1 (MSI-immune, 14 %), CMS2 (canonical, 37 %), CMS3 (metabolic, 13 %) and CMS4 (mesenchymal, 23 %), with a residual unclassified group (mixed features, 13 %). Molecular attributes and expression signatures for each CMS group are indicated. (*CIMP* CpG Island methylator phenotype, *CIN* chromosomal instability, *C’* complement activation signature, *CMS* consensus molecular subtypes, *dMMR* defective mismatch repair, *MLH1-sil* MLH1 silencing by promoter hypermethylation, *MSI* microsatellite instability, *MSS* microsatellite stability, *SCNA* somatic copy number alteration, *POLE* DNA polymerase epsilon (or *D1*, Delta 1)).

CRC is not a homogenous disease, but can be classified into different subtypes, which are characterised by specific molecular and morphological alterations. A major feature of CRC is genetic instability that can arise by at least two different mechanisms. The most common (around ∼84 % of sporadic CRC) is characterised by chromosomal instability (CIN), with gross changes in chromosome number and structure including deletions, gains, translocations and other chromosomal rearrangements. These are often detectable as a high frequency of DNA somatic copy number alterations (SCNA), which are a hallmark of most tumours that arise by the adenoma-carcinoma sequence [5]. Previous molecular genetic studies have associated CIN with inactivating mutations or losses in the Adenomatous Polyposis Coli (*APC)* tumour suppressor gene, which occur as an early event in the development of neoplasia of the colorectum in this sequence. The second group (around ∼13–16 % of sporadic CRC) are hypermutated and show microsatellite instability (MSI) due to defective DNA mismatch repair (MMR), often associated with wild-type *TP53* and a near-diploid pattern of chromosomal instability (Fig. 1) [6]. Furthermore, CpG island methylation phenotype (CIMP) is a feature that induces epigenetic instability by promotor hypermethylation and silencing of a range of tumour suppressor genes, including *MLH1*, one of the MMR genes [7]. This review provides an overview of the integrated molecular and transcriptomic patterns in CRC, including new insights from The Cancer Genome Atlas (TCGA) project [8] and the Consensus Molecular Subtype (CMS) Consortium [9].

## Chromosomal instability is linked to abnormalities of the WNT signalling pathway

CIN tumours usually arise as a consequence of a combination of oncogene activation (e.g. *KRAS*, *PIK3CA*) and tumour suppressor gene inactivation (e.g. *APC*, *SMAD4* and *TP53*) by allelic loss and mutation, which go along with changes in tumour characteristics in the adenoma to carcinoma sequence, as first described by Fearon and Vogelstein in 1990 [10]. A key early event in this pathway is hyperactivation of the WNT signalling pathway, usually arising from mutations of the *APC* gene. Abnormalities of the WNT pathway characterise the majority of sporadic colorectal cancers, as well as tumours that arise in FAP patients [11]. Over 80 % of adenomas and CRC exhibit APC mutations and a further 5–10 % are showing mutations or epigenetic changes in other WNT signalling components (e.g. β-catenin) that equally result in hyperactivation of the WNT pathway [12–14]. APC is an important negative regulator of the WNT pathway, being a component of the Axin-APC degradosome complex that promotes the proteasomal degradation of the WNT effector β-catenin. If this complex is defective as a consequence of mutational inactivation of APC, excess β-catenin accumulates within the cytoplasm and translocates into the nucleus where it operates a transcriptional switch leading to activation of *MYC* and many other genes [15]. Perturbation of the WNT pathway leads to a dysregulation of proliferation and differentiation with the development of dysplastic crypts, which progress to adenomas with increasing grade of dysplasia owing to loss of other tumour suppressor genes. The transition from adenoma to invasive carcinoma is usually associated with mutation and/or loss of the *TP53* tumour suppressor gene.

## Defective DNA mismatch repair leads to microsatellite instability in sporadic hypermutated cancers and Lynch syndrome cancers

Lynch syndrome (LS), also previously known as hereditary non-polyposis colorectal cancer syndrome (HNPCC), is a syndrome of inherited susceptibility to cancers of several organs, primarily the large bowel, with the next most frequently affected being the endometrium. Moreover, there is also an increased risk of adenocarcinomas of the ovary, stomach, small intestine, transitional cell tumours of ureter and renal pelvis, skin neoplasms (sebaceous tumours and keratoacanthomas), and brain gliomas, amongst others. Development of a neoplasm involves inheriting and acquiring defects in the DNA MMR system in the neoplastic cells. The syndrome is caused by dominant inheritance of a mutant MMR gene (mostly either *MSH2* or *MLH1*), with all somatic cells containing one mutated and one wild-type MMR allele. During tumour formation, there is inactivation of the second MMR allele, by mutation, deletion or promoter methylation (in the case of the *MLH1* gene), such that the neoplastic cell has inactivated both MMR alleles. In contrast, in sporadic colorectal cancers with defective mismatch repair, the mechanism is almost always (>95 %) promoter hypermethylation of both alleles of the *MLH1* gene, thus silencing MLH1 expression and crippling the MMR pathway [16–20]. The selective pressure for defective mismatch repair within a neoplasm appears to be due to the reduced susceptibility to apoptosis induced by mismatch-related DNA damage [21–23].

**Fig. 2:**
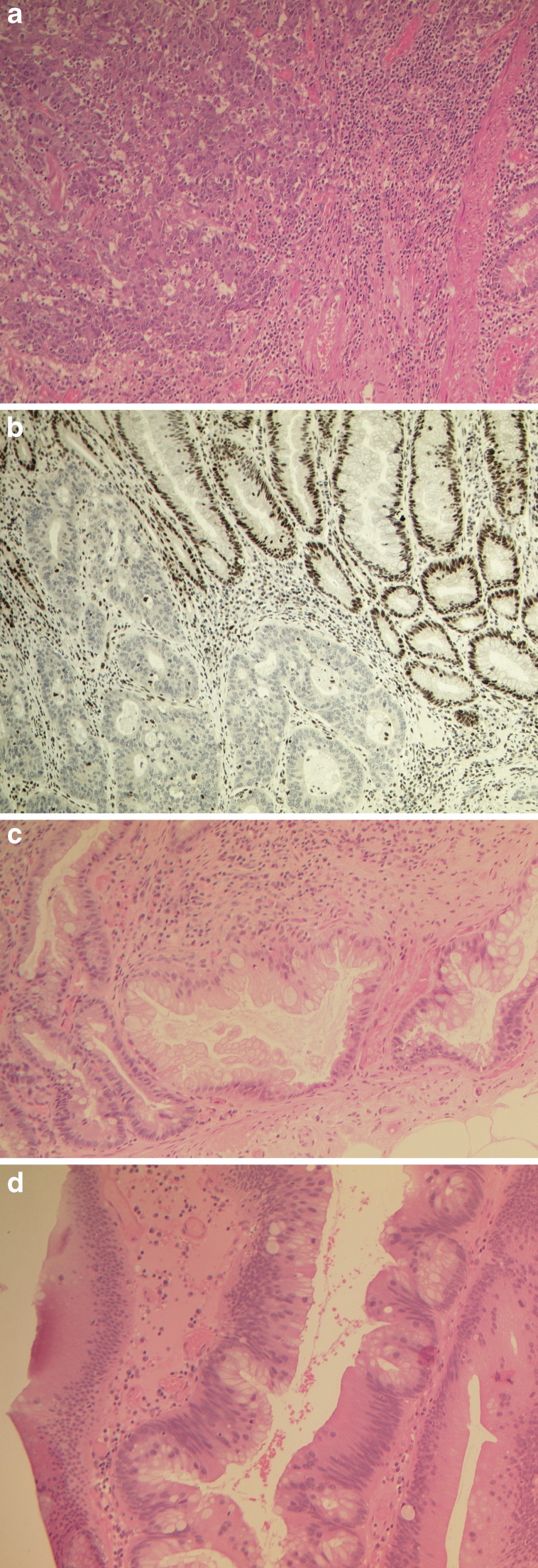
Integration of morphological and molecular features of colorectal cancer, including the serrated precursors sessile serrated adenoma/polyp and traditional serrated adenoma. **a** Poorly differentiated colorectal cancer (*on the left*) of CMS1 (MSI-immune) with prominent tumour-infiltrating lymphocytes (TILs) and underlying lymphocytes within the submucosa with adjacent muscularis mucosae and crypt bases (*on the right*). **b** Immunohistochemical stain for MLH1 showing loss of expression of MLH1 protein in the adenocarcinoma (*bottom left*) with positive staining for MLH1 in the overlying adenoma (*top right*) and adjacent lymphoid and stromal cells. **c** Sessile serrated adenoma/polyp showing a high-power view of the bases of dilated and serrated crypts with boot-shaped architecture and horizontal growth along the top of the muscularis mucosae, with mild nuclear enlargement but no dysplasia. **d** Traditional serrated adenoma showing a high-power view of an elongated dysplastic crypt with small lateral ectopic budding crypts, projecting at 90° to the main axis of the long crypt. The nuclei are elongated, displaying a pencillate pattern of low-grade dysplasia. (All photomicrographs taken at ×100 magnification)

LS colorectal cancers are adenocarcinomas in type, often poorly differentiated or sometimes undifferentiated, occasionally with a dyscohesive appearance. They have prominent tumour-infiltrating lymphocytes and peritumoural Crohns-like lymphoid cell aggregates (Fig. 2) and arise more often in the proximal than in the distal bowel. The major affected genes in LS are *MSH2* and *MLH1*, accounting for 40–45 % LS families each, with the others being mostly due to MSH6 and PMS2 mutations (∼5–10 % LS families each), with rare LS families having other affected genes [18]. The resulting failure to repair DNA replication-associated mismatch errors in these tumour cells produces a high frequency of mutations, either as single base mismatches or in regions of short tandem DNA repeats (the repeat units often being 1–4 bp in length), known as microsatellites. Thus, DNA extracted from such LS tumours shows variation in length (longer and shorter) of a significant proportion of microsatellites, often more than 30 % of those microsatellite markers tested, a phenomenon known as microsatellite instability at high frequency (MSI-H).

Following DNA damage or most commonly following DNA replication-associated mismatch errors, MMR proteins normally recognise both base mismatches and the insertion/deletion loops (IDLs) that occur in repetitive sequences. Recognition of mismatches and single base IDLs involves the heterodimeric complexes of MutS-related proteins MSH2 and MSH6 (known as hMutS-Alpha), whereas IDLs of 2–8 nucleotides are recognised by the complex of MSH2 and MSH3 (known as hMutS-Beta). There is overlap in the specificities of these two complexes and hence some redundancy in their activity. A second type of heterodimeric complex, involving two MutL-related proteins, such as either MLH1 and PMS2 (hMutL-Alpha), or MLH1 and PMS1 (hMutL-Beta), binds to the hMutS complex along with other protein components, so that excision of the recently synthesised error-containing DNA strand occurs and resynthesis of the correct sequence of nucleotides can take place, thus repairing the error [20].

Loss or abnormal expression of the MMR proteins MLH1, MSH2, MSH6 and PMS2, assessed by immunohistochemistry, is standard practice in many pathology laboratories and is used to help identify LS cancers along with MSI typing of tumour DNA [24–26] (Fig. 2). Distinguishing LS colorectal cancers that show loss of MLH1 expression from sporadic MMR-deficient cancers is currently most appropriately performed by detection of the specific mutation *BRAF* V600E, which is found in around 80–90 % of sporadic MSI-H colorectal cancers, but rarely—if ever—in colorectal cancers due to Lynch syndrome [6, 27–31]. The presence of *MLH1* promoter hypermethylation may be used to distinguish sporadic CRC from Lynch syndrome-associated CRC, but there are interpretative problems as constitutive *MLH1* promoter methylation may occur, as well as technical challenges of performing this test [19]. In addition to *MLH1*, there are a number of other genes displaying DNA promoter hypermethylation changes, sometimes referred to as CIMP-genes, but there is some disagreement regarding which are the most reliable CIMP-genes and which tests to use for identification of CIMP tumours [7, 14, 32].

## Correlation of molecular pathways with serrated morphology

In addition to CRC development via the well-described adenoma-carcinoma sequence, it is estimated that about 10–20 % of carcinomas may develop via a different sequence of morphological changes, known as the serrated pathway. While the majority of serrated polyps (80–90 %) can be characterised as hyperplastic polyps, which are considered benign bystander lesions, a subset of serrated lesions can progress to colorectal carcinoma. The two premalignant precursor lesions are traditional serrated adenomas (TSA) and sessile serrated adenomas/polyps (SSA/P) (termed sessile serrated adenomas or alternatively sessile serrated polyps, previous European recommendations have also suggested the term sessile serrated lesions) [33, 34] (Fig. 2).

Cancers arising via the two serrated pathways are heterogeneous in terms of molecular patterns and cannot easily be classified based on characteristic mutations, but rather by specific morphologic changes. A common feature of the serrated pathways is mutations in *KRAS* or *BRAF*, leading to hyperactivation of the MAPKinase pathway. Furthermore EphB2 can be downregulated by genomic loss or promoter methylation, also resulting in MAPK hyperactivation [33, 35, 36]. The characteristic morphological features of the traditional serrated pathway such as architectural dysplasia with ectopic budding crypt formation and epithelial serrations are likely to be linked with these molecular alterations that result in hyperproliferation and inhibition of apoptosis [33, 37–39].

TSAs are more often diagnosed in the left colon. They frequently (∼80 %) have *KRAS* mutations or less often (20–30 %) *BRAF* mutations and are microsatellite stable (MSS) or MSI-L. They are diagnosed based on characteristic cytology (eosinophilic cytoplasm, central, elongated hyperchromatic nuclei) and slit-like epithelial serrations with ectopic crypt formation and may progress to adenocarcinoma (traditional serrated pathway) [35, 40].

SSA/P frequently occur in the right colon, and they tend to have *BRAF* mutations (∼80 %). CIMP is an early feature of SSA/P and often leads to MSI, related to *MLH1* promoter hypermethylation. Also, *MTMG* can be silenced by promoter methylation, which on its own results in an MSI-L phenotype. SSA/P are characterised by abnormally shaped (boot, inverted-anchor, J, L or inverted T) crypts or horizontal growth along the muscularis mucosae, with crypt dilatation and serration extending down to the crypt base [41]. These architectural changes (without genuine dysplasia) are the hallmark of SSA/P and are believed to result from a displacement of the maturation zone [33, 41, 42]. SSA/P may progress to serrated or mucinous adenocarcinomas (sessile serrated pathway).

Colorectal cancers arising via the serrated pathways have been recognised as a distinct subtype overlapping with CIN and MSI tumours by molecular profiling, and are strongly associated with poor prognosis and therapy resistance. Since EMT and matrix remodelling proteins are upregulated in these lesions, it was hypothesised that this predisposes CRC developing via the serrated pathways to invasiveness and metastasis at an early stage [43]. Subsequent analysis revealed that MSI, which often develops within SSA/P, resulted in a more favourable prognosis, whereas MSS in carcinomas derived from SSA/P, and more often from TSA, was linked to poor prognosis [35, 36].

## Integrated genomic characterisation of colorectal cancers (TCGA classification)

The TCGA network project collected colorectal tumour samples and corresponding germline DNA samples from 276 patients for exome sequencing of 224 cancers with paired normal samples, along with DNA SCNA analysis, promoter methylation, messenger RNA (mRNA) and micro RNA (miRNA) studies. Ninety-seven samples underwent whole genome sequencing. The clinical and pathological characteristics reflected the typical cross-section of patients with CRC, so this data provides a valuable source of information to gain further insights into the molecular pathology of CRC [8].

The analysis revealed that the bowel cancers could be split into two major groups by mutation rate—non-hypermutated and hypermutated cancers—which by characteristics and frequency match well with the previously discussed CIN and MSI pathways (Fig. 1, Table 1). The hypermutated category was further subdivided in two subgroups. While the majority of tumours in this group (∼13 % of the analysed tumours) were hypermutated cancers due to defective mismatch repair (dMMR) with a high mutation rate of 12–40 mutations/Mb, a small subgroup (∼3 % of the analysed tumours) had an extremely high mutation rate of >40 mutations/Mb and were thus called ultramutated cancers. The dMMR of the hypermutated cancers resulted from acquired hypermethylation of the *MLH1* promoter in almost all cases, leading to the silencing of expression of MLH1 and non-functioning mismatch repair, which is again in accordance with the previously discussed findings. Almost all of these tumours showed CIMP characteristics, with several other specifically tested genes also demonstrating promoter methylation. A small number of cancers showed either inherited (LS/HNPCC) or somatic MMR gene mutations. The ultramutated colorectal carcinomas had an extremely high mutation rate with a characteristic nucleotide base change spectrum with increased C-to-A transversions, resulting from the presence of a mutation that inactivates the proofreading function within the exonuclease domain of the polymerase E (POLE) DNA replicating enzyme, or rarely of POLD1. This resulted in failure to correct the misincorporation of nucleotides during DNA replication or repair by mutant POLE (or D1). Other studies [44, 45] have shown that less than 0.1 % of CRC have inherited mutations at characteristic sites within the exonuclease domain of either *POLE* (p.Leu424Val) or *POLD1* (p.Ser478Asn), which are the basis of the polymerase-proofreading-associated polyposis (PPAP) syndrome that is characterised by increased colorectal adenomas and adenocarcinomas as well as increased risk of endometrial cancer in the case of inherited POLD1 mutations [44]. The group of non-hypermutated cancers with a low mutation rate (<8 mutations/Mb) mostly demonstrated a high SCNA frequency, making up the majority (∼84 %) of colorectal adenocarcinomas that were MSS due to an intact MMR pathway.

**Table 1:** Characteristics of colorectal cancers in TCGA integrated molecular classification

Group	(1a) Ultramutated *POLE* mutant	(1b) Hypermutated dMMR/MSI	(2) CIN/SCNA-high, MSS
Mutation rate	++++	+++	+
Somatic copy number alterations	+/−	+	+++
Key molecular/genetic abnormality	*POLE* EDM proofreading mutation	Defective MMR/MLH1 promoter hypermethylation	Variety of mutated cancer genes; WNT pathway activation (mostly by APC mutation/inactivation)
Predominant histological type	Moderately differentiated adenocarcinoma	Mucinous, or signet ring, or poorly differentiated adenocarcinoma	Moderately differentiated adenocarcinoma
Proportion of all colorectal carcinomas	∼3 %	∼13 %	∼84 %
Prognosis	Good (more data required)	Good/poor after relapse	Good-poor (depending on other characteristics)

*CIN* chromosomal instability, *POLE* DNA polymerase epsilon, *EDM* exonuclease domain mutant, *SCNA* somatic copy number alteration, *MMR* mismatch repair, *MSI* microsatellite instability

Ultramutated and hypermutated cancers were combined into a single group and compared with the low mutation rate MSS tumours. Overall, 32 genes were recurrently mutated and after removal of non-expressed genes, there were 15 and 17 recurrently mutated genes in the hypermutated and non-hypermutated bowel cancer groups, respectively. The significantly mutated genes in the hypermutated cancers included *ACVR2A* (63 %), *APC* (51 %), *TGFBR2* (51 %), *BRAF* (46 %), *MSH3* (40 %), *MSH6* (40 %), *MYOB1* (31 %), *TCF7L2* (31 %), *CASP8* (29 %), *CDC27* (29 %), *FZD3* (29 %), *MIER3* (29 %), *TCERG1* (29 %), *MAP7* (26 %), *PTPN12* (26 %) and *TP53* (20 %). The genes that were recurrently mutated in the non-hypermutated MSS colorectal cancers included mutations in *APC* (81 %), *TP53* (60 %), *KRAS* (43 %), *TTN* (31 %), *PIK3CA* (18 %), *FBXW7* (11 %), *SMAD4* (10 %), *NRAS* (9 %), *TCF7L2* (9 %), *FAM123B*, also known as *WTX*, (7 %), *SMAD2* (6 %), *CTNNB1* (5 %), *KIAA1804* (4 %), *SOX9* (4 %), *ACVR1B* (4 %), *GPC6* (40 %) and *EDNRB* (3 %). The tumour suppressor genes *ATM* and *ARID1A* showed a disproportionately high percentage of nonsense or frameshift mutations. The *KRAS* and *NRAS* mutations were activating oncogenic mutations at codons 12, 13 and 61, and the *BRAF* mutation was the classical V600E activating mutation, whereas the other genes almost entirely had inactivating mutations.

Colonic and rectal cancers were combined for the analysis of the non-hypermutated MSS group, as they showed no distinguishable molecular differences. SCNA patterns in non-hypermutated MSS tumours confirmed the previously well-documented [5] chromosomal arm-level changes of significant gains of 1q, 7p, 7q, 8p, 8q, 12q, 13q, 19q and 20p, and significant deletions of 1p, 4q, 5q, 8p, 14q, 15q, 17p (includes *TP53*) and 17q, 18q (includes *SMAD4*), 20p and 22q. Hypermutated MSI cancers had far fewer SCNAs, but a similar pattern of chromosomal arm gains and losses. There were 28 recurrent deletion peaks that included the genes *FHIT*, *RBFOX1*, *WWOX*, *SMAD4*, *APC*, *PTEN*, *SMAD3* and *TCF7L2*. Other studies have identified *PARK2* as another recurrently deleted gene on chromosome 6 in around a third of CRCs [46]. A chromosomal translocation generating a gene fusion of *TCF7L2* and *VT11A* was seen in 3 % of CRC and also *NAV2*-*TCF7L1* fusion in three cancers. Focal amplifications were seen affecting *MYC*, *ERBB2*, *IGF2*, *USP12*, *CDK8*, *KLF5*, *HNF4A*, *WHSC1L1*/*FGFR1* and gains of *IRS2* [47].

The most frequently altered pathways by gene mutations, deletions, amplifications and translocations were activation of the WNT, MAPK and PI3K signalling pathways, and deactivation of the TGF-β and P53 inhibitory pathways, which may be relevant for targeted therapies. The WNT signalling pathway was activated in 93 % of non-hypermutated and 97 % of hypermutated cancers, involving biallelic inactivation of *APC* or activation of *CTNNB1* in over 80 % of tumours, together with changes to many other genes involved in regulation of the WNT pathway (*TCF7L2*, *DKK*, *AXIN2*, *FBXW7*, *ARID1A*, *FAM123B*, *FZD10* and *SOX9*). Alterations affecting either the MAPK (*ERBB2*, *RAS* genes, *BRAF*) or PI3K (*PIK3CA*, *PIK3R1*, *PTEN*, *IGF2*, *IRS2*) signalling pathways were relatively common, often showing patterns of mutual exclusivity of gene mutations (for *RAS* and *BRAF* or for *PIK3CA*, *PIK3R1* and *PTEN*). The TGF-β pathway was deregulated by alterations to *TGFBR1*, *TGFBR2*, *ACVR2A*, *ACVR1B*, *SMAD2*, *SMAD3* and *SMAD4* in 27 % of non-hypermutated MSS tumours and 87 % of hypermutated cancers. The P53 pathway was affected by mutations to *TP53* (60 %) and *ATM* (7 %) in a near mutually exclusive pattern in non-hypermutated MSS bowel cancers. An integrated data analysis showed that nearly all tumours displayed dysregulation of *MYC* transcriptional targets as a result of *MYC* activation by activated WNT signalling and/or dysregulation of TGF-β signalling, indicating an important role for *MYC* in colorectal cancer. Using CRC resection data on stage, nodal status, distant metastasis and vascular invasion, some molecular changes were associated with aggressive features including those affecting *SCN5A*, *APC*, *TP53*, *PIK3CA*, *BRAF* and *FBXW7* as well as altered expression of some miRNAs. Potential therapeutic approaches suggested by the TCGA classification are targeting of IGF2, IGFR, ERBB2, ERBB3, MEK, AKT and mTOR proteins as well as possible WNT pathway inhibitors.

## Colorectal cancer gene expression profiling (CMS Classification)

Early attempts at gene expression profiling in order to stratify CRC were made by several groups, but showed little agreement with each other, suggesting different categories, and did not lead to a useful single consistent classification system [43, 48–53]. Subsequently, an international expert consortium [9] recently reached an agreement that describes four consensus molecular subtypes (CMS) after analysis of 18 different CRC gene expression datasets, including data from TCGA in conjunction with molecular data on mutations and SCNAs for a subset of the samples (Fig. 1).

CMS1 (MSI-immune, 14 %) CRC were hypermutated due to defective DNA mismatch repair with MSI and MLH1 silencing and accordingly CIMP-high with frequent *BRAF* mutations, while having a low number of SCNAs. This equates with the previously well-characterised sporadic MSI CRC subgroup. Gene expression profiling furthermore revealed evidence of strong immune activation (immune response, PD1 activation, NK cell, Th1 cell and cytotoxic T cell infiltration signatures) in CMS1, consistent with pathological descriptions of prominent tumour-infiltrating CD8+ cytotoxic T lymphocytes. Patients with the CMS1 subtype had a very poor survival rate after relapse.

The majority of CRC previously described as CIN was split into three subcategories based on transcriptomic profiling, which consequently were all characterised by high levels of SCNAs. CMS2 (canonical, 37 %) CRC predominantly displayed epithelial signatures with prominent WNT and MYC signalling activation, and more often displayed loss of tumour suppressor genes and copy number gains of oncogenes than the other subtypes. CMS2 patients had a better survival rate after relapse compared with the other subtypes. The CMS3 (metabolic, 13 %) subtype had fewer SCNAs and contained more hypermutated/MSI samples than CMS2 and CMS4, along with frequent *KRAS* mutations and a slightly higher prevalence of CIMP-low. Gene expression analysis of CMS3 found predominantly epithelial signatures and evidence of metabolic dysregulation in a variety of pathways. The CMS4 subtype (mesenchymal, 23 %) CRC showed increased expression of EMT genes and evidence of prominent transforming growth factor-β activation, with expression of genes implicated in complement-associated inflammation, matrix remodelling, stromal invasion and angiogenesis. Patients with the CMS4 subtype had a worse overall survival and worse relapse-free survival than patients of the other groups. Finally, there were some samples with mixed features (13 %) that possibly represent either a transition phenotype or intratumoural heterogeneity.

This CMS classification system has been suggested by the authors to be the most robust classification system currently available for CRC based on biological processes related to gene expression patterns and is suggested as a basis for future clinical stratification in trials and other studies with potential for subtype-based targeted interventions, although further studies are required to validate this assertion.

## Conclusion

In conclusion, integration of wide-ranging molecular data has generated two systems of classification of colorectal cancers (Fig. 1, Table 1). (A) TCGA classification—tumours with a very high mutation rate which can be further subdivided into either (1a) ultramutated colorectal cancers (∼3 %) with DNA polymerase epsilon (POLE) proofreading domain mutations, or (1b) hypermutated colorectal cancers (∼13 %) with microsatellite instability due to defective mismatch repair; and (2) colorectal cancers (∼84 %) with a low mutation rate but a high frequency of DNA SCNAs. (B) The CMS classification describes four CMS groups—CMS1 (MSI-immune activation, 14 %), CMS2 (canonical, 37 %), CMS3 (metabolic, 13 %) and CMS4 (mesenchymal, 23 %), with a residual unclassified group (mixed features, 13 %). Further research is required to develop more easily applicable molecular tests, such as low-coverage high-throughput sequencing for DNA SCNA analysis and/or cancer gene panel mutation detection, and preferably easily applicable and useful immunohistochemical markers for these CMS subdivisions. Analysis of expression of the MMR proteins and/or MSI testing is currently efficient at identifying the group of defective mismatch repair MSI tumours (CMS1). Both classification systems agree on identification of this dMMR/MSI group, which has recently been shown to respond well to immune checkpoint blockade (antibodies to PD-1) that activates cytotoxic T cell attacks on tumour cells, which is suggested to be related to the large numbers of neo-antigens generated by dMMR [54, 55]. A straightforward and routinely applicable molecular test using PCR and sequencing for identification of POLE (and POLD1) proofreading mutations associated with ultramutated cancer may be performed in molecular pathology laboratories, although in the future a mutation-specific POLE antibody for immunohistochemistry may be developed to aid routine subclassification. Ultramutated cancers are likely to generate higher levels of neo-antigens and may also respond well to immune checkpoint blockade therapy. Selected transcript expression profiling kits for CMS classification may be required for application of this system. Both classification systems have been proposed to allow better prognostication and are potentially important for future use in clinical trials and for multidisciplinary team discussions about post-surgical adjuvant treatment, including immune checkpoint blockade.
